# Examination of Lead and Cadmium in Water-based Paints Marketed in Nigeria

**DOI:** 10.5696/2156-9614-6.12.43

**Published:** 2016-12-22

**Authors:** Ajoke F. I. Apanpa-Qasim, Adebola A. Adeyi, Sandeep N. Mudliar, Karthik Raghunathan, Prasant Thawale

**Affiliations:** 1 Department of Chemistry, University of Ibadan, Ibadan-Nigeria; 2 CSIR-National Environmental Engineering and Research Institute, Nagpur, India; 3 CSIR-Central Food Technological Research Institute, Mysore, India

**Keywords:** heavy metals, paints, exposure, lead, cadmium, ICP-OES

## Abstract

**Background.:**

In spite of the availability of substitutes for lead and cadmium compounds in paints, manufacturers continue to produce paints with high levels of these metals. As the population continues to grow and there is a continued shift from oil-based to water-based paints, the sales and use of these paints will increase the exposure of humans and the environment to these metals.

**Objectives.:**

We measured the levels of lead (Pb) and cadmium (Cd) in 174 paint samples marketed in Lagos and Ibadan, Nigeria. Paint samples from different manufacturers registered with and without Standards Organization of Nigeria (SON) were considered.

**Methods.:**

Samples were acid digested using a microwave digester and the levels of the elements were determined using inductively coupled plasma optical emission spectroscopy (ICP-OES).

**Discussion.:**

The levels of Cd and Pb (dry weight) in all samples ranged from 98–1999 μg/g and 170–3231 μg/g, respectively. All the samples were above the permissible limits of 90 ppm of the US Consumer Product Safety Commission and 100 ppm limit of the European Union (EU) for Pb and Cd in paint.

**Conclusions.:**

We concluded that water-based paints marketed in Nigeria still contain substantial amounts of lead and cadmium which are detrimental to human health and the entire ecosystem. These metals are among the EU priority metals due to the increased risk of occupational exposure to humans and vulnerable groups such as children.

## Introduction

Environmental health hazards caused by heavy metals exposure are a continued threat to human health, particularly in developing countries.^1^ There is increasing awareness of consumer products with high heavy metal content. Heavy metals such as lead, cadmium, arsenic and so on are constituents of pigments which are added to paint formulations to increase brightness and longevity.^2^ Paints and coatings play an indispensable role in household appliances, buildings, cars, ships, aeroplanes, computers, furniture, and circuit boards.^3^

Lead (Pb) is a toxic heavy metal even at very low levels of human exposure. Lead can cause central nervous system damage and is an important disease-causing agent in terms of environmental contribution to the total global burden of disease as measured in disability adjusted life years.^4^ Lead can also damage the kidney, liver and reproductive system, basic cellular processes and brain function. Its effect on the human body can be both acute or chronic depending on dose and exposure scenarios. Toxic symptoms can include anemia, insomnia, headache, dizziness, irritability, muscle weakness, hallucination and renal damage.^5^ Lead accumulates in the skeleton and its mobilization from bones during pregnancy and lactation causes exposure to the fetus and breast fed infants.^6–8^

Cadmium (Cd) is a major pollutant that causes a range of pathological alterations.^9–12^ It is used in various industrial activities. Major industrial applications of cadmium include the production of alloys, pigments, and batteries.^13^ Cadmium is a severe pulmonary and gastrointestinal irritant, and can be fatal if inhaled or ingested. It accumulates primarily in the kidneys and has a long biological half-life of 10–35 years in humans. The primary effect of cadmium is kidney damage and bone fragility.^14–16^ There is evidence that cadmium is carcinogenic through the inhalation route, and humans are exposed to cadmium by inhalation and ingestion.^17^,^18^

Both Pb and Cd are widely distributed in the environment (air, soil, surface and ground water, sediment, dust, food, paint) and in biological systems; and occur both naturally and through human activities.^5^,^13^,^19–23^ Unlike organic contaminants, lead and cadmium are not biodegradable and tend to accumulate in living organisms.^5^ They are readily transferred through the food chain and are not known to serve any essential biological function.

**Abbreviations**
*Cd*: Calcium
*Pb*: Lead

Several studies have been conducted on metals in consumer products. Cadmium has been analyzed in cosmetics and personal care products, toys, children's jewelry, and decorative paints.^24–37^ Global studies on lead in new decorative paints conducted by Toxic Link showed that 68.5% of the 232 enamel samples collected from eleven countries had lead concentrations above the 90 ppm permissible limit of the US Consumer Product Safety Commission and 64.6% of the samples had lead concentrations above 600 ppm, which is the previous permissible limit of the US Consumer Product Safety Commission and the regulatory standard of Argentina, Chile and Uruguay.^38^ In addition, a study by Clark et al. showed a total of 57 (73%) out of 78 new enamel household paints had at least one sample with a lead concentration above or equal to 600 ppm.^39^

Various countries have initiated laws that regulate or ban the use of hazardous chemicals and substances in paint with varying degrees of compliance.^40^,^41^ There are regulations in place in Nigeria stipulating that paints should contain less than 90 ppm (dry weight) of total lead and 100 ppm of cadmium, but these limits have not been enforced.^42–44^ Thus, we conducted a survey of decorative paints currently sold in Nigeria using Lagos and Ibadan as case studies. We assessed the levels of lead and cadmium in water-based paints so as to ascertain the levels of exposure to these metals from paint products.

## Methods

### Sample Collection

Paint samples were purchased in popular paint markets in Ibadan and Lagos, Nigeria based on color availability and the most commonly used water-based paints. Two samples each of the same color produced by 14 different manufacturers were collected. Six manufacturers were registered with the Standards Organization of Nigeria (SON), and 8 manufacturers were unregistered. A total of 12 different paint colors were collected, as shown in Table 1. Samples were stored in air-tight plastic containers and analyzed at the Council of Scientific and Industrial Research-National Environmental Engineering Research Institute Laboratory, Nagpur—Maharashtra, India.

**Table 1:** Paint Samples Collected in Lagos and Ibadan, Nigeria

***Sample Number***	**Manufacturer**	**Number of paint colours collected**	**Total number of paint samples collected**	**Standards Organization of Nigeria registration**
**1**	A	9	18	Yes
**2**	B	10	20	Yes
**3**	C	9	18	Yes
**4**	D	5	10	Yes
**5**	E	5	10	Yes
**6**	F	6	12	Yes
**7**	G	8	16	No
**8**	H	5	10	No
**9**	I	5	10	No
**10**	J	7	14	No
**11**	K	6	12	No
**12**	L	4	8	No
**13**	M	4	8	No
**14**	N	4	8	No

### Sample Preparation and Pre-treatment

Approximately 5 mL of the paint samples were spread on glass slides using a different brush for each sample to avoid cross contamination. The glass slides were placed in an oven at 120°C for 2 hours. About 1.0 g each of the dried paint film was weighed into closed Teflon vessels and digested in a closed microwave digestion system using 10 mL of 70% nitric acid and 3 mL of 98% sulphuric acid. Samples were analyzed according to the standard procedures for digestion of very difficult samples.^45^ Digestates were then filtered and analyzed using inductively coupled plasma-optical emission spectrometry (Thermo Scientific iCAP 6300 Duo).

Sample blanks were also prepared alongside the samples. Dilutions were performed when necessary for the samples. Working standard solutions of 1–1000 μg/g were prepared from a multielement standard. A recovery study was carried out and recoveries of 80% for Pb and 110% for Cd were obtained.

### Instrument Operation Conditions

#### Digestion of Paint Samples

A MARS 6 microwave reaction system was used for sample digestion. Samples were weighed into Teflon vessels and digested at 200°C, 25 mins ramp time, 30 mins holding time, and 1000 watt power in one cycle digestion. The temperature guard of the instrument was set to not exceed 210°C. Cooling was automatically carried out by the instrument to 30°C for another 30 minutes.

#### Inductively Coupled Plasma Optical Emission Spectrometer

Digested samples were analyzed using Thermo Fisher Scientific inductively coupled plasma optical emission spectrometry (model iCAP 6300 Duo) which was coupled with an auto sampler CETAC ASX-52, and spectrometer (Echelle type) equipped with a simultaneous charge injection device detector measuring wavelengths from 166.00 nm to 847.00 nm. The operating condition of ICP-OES was 1150 W RF power, 15 L/min plasma flow, 50 rpm pump rate, 0.5 L/min auxiliary gas flow, and 0.5 L/min nebulizer gas flow. The carrier gas of the instrument was ultra-pure argon.

## Results

The levels of Cd and Pb in the paint samples collected in Lagos and Ibadan, Nigeria are presented in the Supplemental Material, while Table 2 shows a comparison of the concentrations of Pb in the paint samples obtained in this study with previous studies reported in the literature.

**Table 2:** Comparison of the Present Study with Other Studies Reported in the Literature for Lead in Paint Samples

***Countries***	**Samples**	**Number of Samples**	**Average concentration**	**Maximum concentration**	**Percentage of samples with concentrations ≥ 90 ppm^a^**	**Percentage of samples with concentrations ≥ 600 ppm^b^**	**Reference number**
**Nigeria**	water-based	174	811	3231	100	72	This study
**Nigeria**	water-based	8	86	516	5	none	46
	oil-based	11	45	159	11	none	
**Nigeria**	oil-based	25	14500	50000	NA	96	47
**Cameroon**	oil-based	61	NA	500000	66	64	48
**Brazil**	oil-based	20	5600	5900	35	30	41
**Sri Lanka**	water-based	11	4177	45743	10	10	38
	oil-based	19	25210	137325	68	68	
**Philippines**	water-based	10	11	40	none	none	38
	oil-based	15	28354	189163	67	60	
**Thailand**	water-based	10	3	15	none	none	38
	oil-based	17	61893	505716	47	47	
**Tanzania**	water-based	6	22	40	none	none	38
	oil-based	20	14537	120862	100	95	
**South Africa**	oil-based	29	19862	195289	65	62	38
**Nigeria**	water-based	7	8458	34598	100	100	38
	oil-based	23	36989.5	129837	100	100	
**Senegal**	water-based	9	5.5	29	none	none	38
	oil-based	21	5866	29717	86	76	
**Belarus**	water-based	8	58	418	none	none	38
	oil-based	22	5558	59387	82	68	
**Mexico**	water-based	10	6	16	none	none	38
	oil-based	20	51860	163812	100	100	
**Brazil**	water-based	7	10	14	none	none	38
	oil-based	24	15004	170258	42	37	
**India**	oil-based	22	9411	49593	36	36	38
**India**	oil-based	26	16600	134000	42	35	41
**India**	oil-based	5	106000	290000	100	100	49
**India**	water-based	38	NA	140000	38	49	50
	oil-based	31	NA	NA	84	NA	
**India**	oil-based	17	NA	NA	100	NA	51
**India**	oil-based	148	NA	80350	85	NA	52
**Armenia**	oil-based	26	25000	130000	77	77	41
**Kazakhstan**	oil-based	26	15700	71000	81	77	41

Note: NA-not available;

^a^ Present permissible limit of 90 ppm for Pb and

^b^ previous permissible limit of 600 ppm for Pb by the US Consumer Product Safety Commission.

### Concentrations of Lead and Cadmium in Paint Samples by Manufacturer

The levels of Cd and Pb (μg/g, dry weight) in all the samples ranged from 98 1999 and 170–3231, respectively (Supplemental Material). The highest mean concentration of Cd was 1946±75 μg/g obtained in paints produced by manufacturer C, a registered manufacturer. This was followed by 1877±22 and 1845±24 μg/g in products produced by manufacturer L (unregistered) and C, respectively, while the lowest concentration was 98.5±1 μg/g in paints produced by manufacturer K, an unregistered manufacturer. The highest mean concentration of Pb was obtained in paints produced by manufacturer H (3117±161 μg/g), an unregistered manufacturer. This was followed by manufacturer L (3013±16 μg/g) and (2121±19 μg/g) from the same manufacturer, while the lowest concentration, 173±4 μg/g, was found in paints produced by manufacturer K.

The order of metal concentrations in the paint samples with respect to manufacturers was: **Cd**: M > A > L> E > F > J > C > G > D > N > B > I > H >K; and **Pb**: L > N > A > M > H > I > C > E > G > J > B > K> D > F.

The lowest concentrations of Cd and Pb were found in paint samples produced by unregistered and registered manufacturers, respectively. The lowest mean Cd and Pb concentrations (98.5±1 μg/g and 186±1 μg/g) were found in products produced by manufacturers K and F, respectively.

### Concentrations of Lead and Cadmium in Paint Samples by Color

**Figure 1:**
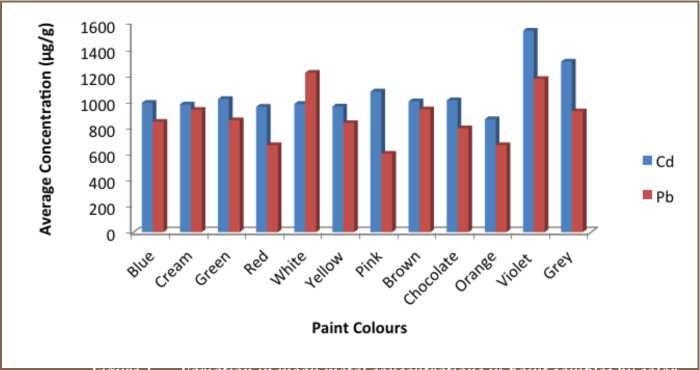
Variation in mean metal concentrations in paint samples by color.

The range of levels of Cd and Pb in all the paint samples with respect to color was 98–1999 μg/g in red color and 170 (green)–3231 μg/g (cream) (*Supplemental Material*), respectively. Variation in mean metal concentrations with respect to color is presented in Figure 1. The highest Cd concentration was in red paint (1999 μg/g), followed by green (1893 μg/g) and red (1893 μg/g) colored paint products. The highest Pb concentration was in cream colored paint (3231 μg/g) (S 1) produced by manufacturer H, followed by green (3024 μg/g) produced by manufacturer L and cream (3003 μg/g) (S 2) also produced by manufacturer H. The highest mean Cd concentration was found in red colored paints (1946±75 μg/g), followed by pink (1845±24 μg/g) and cream (1813 ±28 μg/g), while the lowest concentration was in red (98.5±1 μg/g) colored paints. The highest mean Pb concentration was in cream (3117 ±161 μg/g) paints produced by manufacturer H, followed by green (3013 ±16 μg/g) and blue (2121 ±19 μg/g), both produced by manufacturer L while the lowest concentration was also in green paints (173±4 μg/g) produced by manufacturer K.

The order of Cd and Pb in the paint samples collected with respect to colors was: **Cd**: violet > grey > yellow > pink > green > chocolate > brown > blue > white > cream > red > orange; and **Pb**: violet > brown > cream > grey> green > blue > yellow > chocolate > white > orange > red > pink.

Only one manufacturer (A) out of the fourteen manufacturers considered in this study produced violet colored paints, which ranked first with respect to the levels of Cd and Pb. In addition, only two manufacturers (B and A) produced grey colored paints, which also ranked second and fourth with respect to the levels of Cd and Pb, respectively.

## Discussion

In all the paint samples analyzed in this study, the levels of Pb was above the 90 ppm permissible limit of the US Consumer Product Safety Commission, which came into effect on August 14, 2009, while the levels of Cd were above the 100 ppm permissible limit of the European Union (EU), which came into effect on March 7, 2016.^42^,^43^ The concentrations of Cd and Pb in all of the 174 samples collected in Lagos and Ibadan, Nigeria were above the permissible limits of the EU and US Consumer Product Safety Commission, respectively (*Supplemental Material and Table 2*). These results could be attributed to the raw materials used in the production of paint samples and weak product regulation and enforcement, as registered manufacturers had the highest mean Cd (M) and Pb (L) concentrations in their products. It has been reported that only about 15 paint companies in Nigeria have met the SON product standard.^44^ The health hazards associated with exposure to lead and cadmium in the domestic environment have been insufficiently studied in developing countries, although their importance as a source of morbidity is widely recognized.^53^ Previous studies in Nigeria have shown that over 70% of children have blood lead levels above 10 μg/dL and that flaking paints are an important exposure source.^54^,^55^ Recently, studies showed that blood lead levels once thought safe are associated with increased risk of death from many causes.^56^

The levels of Pb obtained in this study were higher than those reported in Nigeria, Brazil, Mexico, Belarus, Senegal, Tanzania and the Philippines, but lower than some previous studies in Nigeria, Thailand and Sri Lanka (*Table 2*). However, in most cases, the numbers of samples considered in previous studies were far lower than those reported in this study.

## Conclusions

The present study examined the levels of Cd and Pb in paint samples sold in Lagos and Ibadan, Nigeria. In all the 174 paint samples collected and analyzed, the levels of Cd and Pb were above the permissible limits of 100 ppm and 90 ppm of the EU and US Consumer Product Safety Commission, respectively.

The order of metal concentrations with respect to manufacturers were: **Cd**: M > A > L > E > F > J > C > G > D > N > B > I > H > K; and **Pb**: L > N > A > M > H > I > C > E > G > J > B > K> D > F.

The order of metal concentrations with respect to color were: **Cd**: violet > grey > yellow > pink > green > chocolate > brown > blue > white > cream > red > orange; and **Pb**: violet > brown > cream > grey> green > blue > yellow > chocolate > white > orange > red > pink.

Presently, there are regulations in place in Nigeria that stipulate that paints should contain less than 90 ppm (dry weight) of total lead and 100 ppm of cadmium, but these limits have yet to be enforced, leading to high concentrations of these toxic metals in the paint samples. There is a need for the enforcement of permissible limits in paints produced in Nigeria to safeguard public health and to prevent possible exposure of the population to these metals, which are known to be particularly dangerous to humans.
