# Supplementary material for: Examination of Lead and Cadmium in Water-based Paints Marketed in Nigeria
Source: J Health Pollut. 2016 Dec 22;6(12):43–9. doi: 10.5696/2156-9614-6.12.43 (PMC6221508; doi:10.5696/2156-9614-6.12.43)
Supplement: Supplementary file 1 [file Adeyi_SuppMaterial.docx]

| Supplemental Material 1:  Concentrations (dry weight, µg/g) of Cadmium and Lead in Paint Samples Collected in Lagos and Ibadan, Nigeria | | | | | | | | |
| --- | --- | --- | --- | --- | --- | --- | --- | --- |
| Serial  Number | Code | Primary colour in samples collected | Cd | | Mean ± SD | Pb | | Mean ± SD |
| 1 | A | blue | 1692 | 1578 | 1635±81 | 1317 | 1027 | 1172±205 |
|  |  | brown | 1806 | 1355 | 1581±319 | 1271 | 891 | 1081±269 |
|  |  | chocolate | 1682 | 1798 | 1740±82 | 1116 | 1466 | 1291±248 |
|  |  | grey | 1680 | 1706 | 1693±18 | 1103 | 1314 | 1209±149 |
|  |  | violet | 1669 | 1406 | 1537±186 | 1107 | 1231 | 1169±88 |
|  |  | green | 1567 | 1815 | 1691±175 | 1233 | 1182 | 1208±36 |
|  |  | red | 1705 | 1813 | 1759±76 | 1327 | 1279 | 1303±34 |
|  |  | white | 1602 | 1710 | 1656±76 | 1134 | 1305 | 1220±121 |
|  |  | yellow | 1013 | 1587 | 1300±406 | 1181 | 863 | 1022±225 |
| 2 | B | blue | 377 | 405 | 391±20 | 550 | 568 | 559±13 |
|  |  | cream | 761 | 822 | 792±43 | 593 | 639 | 616±33 |
|  |  | green | 842 | 930 | 886±62 | 625 | 503 | 564±86 |
|  |  | pink | 667 | 632 | 650±25 | 640 | 557 | 599±59 |
|  |  | red | 407 | 769 | 588±256 | 568 | 632 | 600±45 |
|  |  | white | 1231 | 958 | 1095±193 | 581 | 703 | 642±86 |
|  |  | yellow | 1123 | 1216 | 1170±66 | 798 | 832 | 815±24 |
|  |  | brown | 850 | 912 | 881±44 | 743 | 803 | 773±42 |
|  |  | chocolate | 779 | 800 | 790±15 | 604 | 625 | 615±15 |
|  |  | grey | 885 | 930 | 908±32 | 765 | 504 | 635±185 |
| 3 | C | blue | 676 | 851 | 764±124 | 1604 | 1781 | 1693±125 |
|  |  | brown | 627 | 602 | 615±18 | 1602 | 692 | 1147±644 |
|  |  | green | 665 | 584 | 625±57 | 789 | 747 | 768±30 |
|  |  | orange | 764 | 772 | 768±6 | 858 | 883 | 871±18 |
|  |  | pink | 1862 | 1828 | 1845±24 | 501 | 402 | 452±70 |
|  |  | red | 1893 | 1999 | 1946±75 | 524 | 521 | 523±2 |
|  |  | white | 750 | 592 | 671±112 | 976 | 958 | 967±13 |
|  |  | yellow | 767 | 777 | 772±7 | 677 | 667 | 672±7 |
|  |  | cream | 509 | 541 | 525±23 | 650 | 690 | 670±28 |
| 4 | D | blue | 969 | 900 | 934±49 | 406 | 461 | 434±39 |
|  |  | cream | 918 | 902 | 910±11 | 390 | 381 | 386±6 |
|  |  | green | 848 | 860 | 854±9 | 361 | 529 | 445±119 |
|  |  | red | 890 | 888 | 889±1 | 531 | 529 | 530±1 |
|  |  | white | 890 | 891 | 891±1 | 409 | 419 | 414±7 |
| 5 | E | blue | 914 | 901 | 908±9 | 643 | 655 | 649±9 |
|  |  | cream | 1556 | 1499 | 1527±40 | 739 | 725 | 732±10 |
|  |  | green | 1041 | 1191 | 1116±106 | 807 | 799 | 803±6 |
|  |  | white | 960 | 988 | 974±20 | 675 | 687 | 681±9 |

|  | | | | | | | | | |
| --- | --- | --- | --- | --- | --- | --- | --- | --- | --- |
| 6 | F | blue | 841 | 830 | 836±8 | 185 | 187 | 186±1 |  |
|  |  | cream | 836 | 1408 | 1122±405 | 196 | 355 | 276±112 |  |
|  |  | green | 840 | 835 | 838±4 | 195 | 196 | 196±1 |  |
|  |  | red | 830 | 836 | 833±4 | 186 | 189 | 188±2 |  |
|  |  | white | 781 | 825 | 803±31 | 173 | 185 | 179±9 |  |
|  |  | yellow | 1406 | 1731 | 1568±230 | 863 | 791 | 827±51 |  |
| 7 | G | blue | 1004 | 823 | 914±128 | 849 | 785 | 817±45 |  |
|  |  | brown | 928 | 902 | 915±18 | 757 | 734 | 746±16 |  |
|  |  | chocolate | 1038 | 870 | 954±119 | 849 | 694 | 771±110 |  |
|  |  | cream | 1003 | 866 | 935±97 | 791 | 685 | 738±75 |  |
|  |  | green | 843 | 792 | 818±36 | 656 | 568 | 612±62 |  |
|  |  | orange | 897 | 833 | 865±45 | 719 | 659 | 689±42 |  |
|  |  | pink | 928 | 975 | 952±33 | 727 | 767 | 747±28 |  |
|  |  | white | 864 | 790 | 827±52 | 678 | 658 | 668±14 |  |
| 8 | H | blue | 952 | 859 | 906±66 | 486 | 229 | 358±182 |  |
|  |  | cream | 863 | 878 | 871±11 | 3003 | 3231 | 3117±161 |  |
|  |  | green | 448 | 501 | 475±38 | 546 | 511 | 529±25 |  |
|  |  | red | 702 | 698 | 700±3 | 300 | 325 | 313±18 |  |
|  |  | white | 941 | 779 | 860±115 | 312 | 368 | 340±40 |  |
| 9 | I | blue | 872 | 734 | 803±98 | 502 | 568 | 535±47 |  |
|  |  | cream | 902 | 827 | 865±53 | 1451 | 1381 | 1416±50 |  |
|  |  | green | 800 | 788 | 794±9 | 543 | 499 | 521±31 |  |
|  |  | pink | 846 | 787 | 817±42 | 564 | 731 | 648±118 |  |
|  |  | white | 760 | 756 | 758±3 | 1480 | 1589 | 1535±77 |  |
| 10 | J | blue | 1239 | 1138 | 1189±71 | 461 | 504 | 483±30 |  |
|  |  | cream | 745 | 781 | 763±26 | 1466 | 1500 | 1483±24 |  |
|  |  | green | 854 | 1036 | 945±129 | 662 | 526 | 594±96 |  |
|  |  | orange | 1085 | 822 | 954±186 | 651 | 433 | 542±154 |  |
|  |  | pink | 1165 | 1040 | 1103±88 | 706 | 460 | 583±174 |  |
|  |  | red | 834 | 835 | 834±1 | 470 | 500 | 485±21 |  |
|  |  | white | 913 | 940 | 927±19 | 592 | 454 | 523±98 |  |
| 11 | K | blue | 844 | 857 | 851±9 | 312 | 323 | 318±8 |  |
|  |  | cream | 766 | 767 | 767±1 | 199 | 199 | 199±0 |  |
|  |  | green | 791 | 802 | 797±8 | 176 | 170 | 173±4 |  |
|  |  | red | 98 | 99 | 98.5±1 | 1377 | 1351 | 1364±18 |  |
|  |  | white | 799 | 803 | 801±3 | 190 | 178 | 184±9 |  |
|  |  | chocolate | 539 | 541 | 540±1 | 505 | 483 | 494±16 |  |

|  | | | | | | | | |
| --- | --- | --- | --- | --- | --- | --- | --- | --- |
| 12 | L | blue | 1041 | 1102 | 1072±43 | 2107 | 2134 | 2121±19 |
|  |  | cream | 841 | 1051 | 946±149 | 483 | 622 | 553±98 |
|  |  | green | 1893 | 1862 | 1878±22 | 3024 | 3001 | 3013±16 |
|  |  | white | 764 | 900 | 832±96 | 442 | 529 | 486±62 |
| 13 | M | blue | 1805 | 1783 | 1794±16 | 1391 | 1275 | 1333±82 |
|  |  | cream | 1833 | 1794 | 1813±28 | 417 | 492 | 455±53 |
|  |  | green | 1801 | 1579 | 1690±157 | 1328 | 1340 | 1334±8 |
|  |  | white | 1795 | 1690 | 1742±74 | 1387 | 1246 | 1317±100 |
| 14 | N | blue | 832 | 806 | 819±18 | 1182 | 1066 | 1124±82 |
|  |  | cream | 880 | 792 | 836±62 | 1181 | 1772 | 1477±418 |
|  |  | green | 869 | 781 | 825±62 | 1103 | 1297 | 1200±137 |
|  |  | white | 815 | 798 | 807±12 | 813 | 1271 | 1042±324 |
